# Longitudinal Development of Antibody Responses in COVID-19 Patients of Different Severity with ELISA, Peptide, and Glycan Arrays: An Immunological Case Series

**DOI:** 10.3390/pathogens10040438

**Published:** 2021-04-06

**Authors:** Jasmin Heidepriem, Christine Dahlke, Robin Kobbe, René Santer, Till Koch, Anahita Fathi, Bruna M. S. Seco, My L. Ly, Stefan Schmiedel, Dorothee Schwinge, Sonia Serna, Katrin Sellrie, Niels-Christian Reichardt, Peter H. Seeberger, Marylyn M. Addo, Felix F. Loeffler, on behalf of the ID-UKE COVID-19 Study Group

**Affiliations:** 1 Department of Biomolecular Systems, Max Planck Institute of Colloids and Interfaces, Am Muehlenberg 1, 14476 Potsdam, Germany; jasmin.heidepriem@mpikg.mpg.de (J.H.); BrunaMara.SilvaSeco@mpikg.mpg.de (B.M.S.S.); Katrin.Sellrie@mpikg.mpg.de (K.S.); Peter.Seeberger@mpikg.mpg.de (P.H.S.); 2 Division of Infectious Diseases, First Department of Medicine, University Medical Center Hamburg-Eppendorf, 20251 Hamburg, Germany; r.kobbe@uke.de (R.K.); t.koch@uke.de (T.K.); a.fathi@uke.de (A.F.); m.ly@uke.de (M.L.L.); s.schmiedel@uke.de (S.S.); 3 Department of Clinical Immunology of Infectious Diseases, Bernhard Nocht Institute for Tropical Medicine, 20251 Hamburg, Germany; 4 German Center for Infection Research, Partner Site Hamburg-Lübeck-Borstel-Riems, 20251 Hamburg, Germany; 5 Department of Pediatrics, University Medical Center Hamburg-Eppendorf, 20251 Hamburg, Germany; r.santer@uke.de; 6 I. Department of Medicine, University Medical Center Hamburg-Eppendorf, 20251 Hamburg, Germany; dschwing@uke.de; 7 Glycotechnology Laboratory, Center for Cooperative Research in Biomaterials (CIC biomaGUNE), Basque Research and Technology Alliance (BRTA), Paseo de Miramon 182, 20014 Donostia San Sebastián, Spain; sserna@cicbiomagune.es (S.S.); nreichardt@cicbiomagune.es (N.-C.R.); 8 CIBER-BBN, Paseo Miramón 182, 20014 San Sebastián, Spain

**Keywords:** SARS-CoV-2, COVID-19, full proteome, peptide microarrays, glycan microarrays

## Abstract

The current COVID-19 pandemic is caused by the severe acute respiratory syndrome coronavirus-2 (SARS-CoV-2). A better understanding of its immunogenicity can be important for the development of improved diagnostics, therapeutics, and vaccines. Here, we report the longitudinal analysis of three COVID-19 patients with moderate (#1) and mild disease (#2 and #3). Antibody serum responses were analyzed using spike glycoprotein enzyme linked immunosorbent assay (ELISA), full-proteome peptide, and glycan microarrays. ELISA immunoglobulin A, G, and M (IgA, IgG, and IgM) signals increased over time for individuals #1 and #2, whereas #3 only showed no clear positive IgG and IgM result. In contrast, peptide microarrays showed increasing IgA/G signal intensity and epitope spread only in the moderate patient #1 over time, whereas early but transient IgA and stable IgG responses were observed in the two mild cases #2 and #3. Glycan arrays showed an interaction of antibodies to fragments of high-mannose and core *N*-glycans, present on the viral shield. In contrast to protein ELISA, microarrays allow for a deeper understanding of IgA, IgG, and IgM antibody responses to specific epitopes of the whole proteome and glycans of SARS-CoV-2 in parallel. In the future, this may help to better understand and to monitor vaccination programs and monoclonal antibodies as therapeutics.

## 1. Introduction

The novel severe acute respiratory syndrome coronavirus 2 (SARS-CoV-2) was first described in Wuhan, China, in January 2020, as the causative agent of COVID-19 [1]. CoVs were not considered to be highly pathogenic, until the emergence of SARS-CoV [2,3,4] in 2002, and the Middle East respiratory syndrome (MERS)-CoV in 2012 [5]. With SARS-CoV-2, three CoVs have passed the species barriers from animal to human in the last 20 years, causing severe respiratory diseases. Based on their pathogenic and epidemic potential, the World Health Organization (WHO) has classified all three CoVs as priority pathogens to accelerate the development of vaccines and therapeutics to prevent epidemics.

The world is still confronted with the SARS-CoV-2 pandemic. This virus belongs to the *Betacoronavirus* genus of the Coronaviridae family and has genetic similarity with SARS-CoV. Within about one year, more than 114 million people have been infected globally, with more than 2.5 million reported deaths as of 2 March 2021 [6]. The infection presents with different symptoms and a wide spectrum of severity [7]. Some patients only experience very mild symptoms like a cough, while others show a very severe form of the disease that leads to bilateral pneumonia.

An efficient countermeasure to limit an outbreak includes specific and sensitive diagnostics. Polymerase chain reaction (PCR) is used to measure SARS-CoV-2 particles, whereas antibodies are measured by enzyme linked immunosorbent assay (ELISA), the gold standard for the detection of SARS-CoV-2 specific antibodies. These tests mainly rely on the binding of serum antibodies to the SARS-CoV-2 spike glycoprotein (S) [8]. The advantage of ELISAs is their simplicity and standardized protocol. A disadvantage is the limitation in sensitivity and specificity, since they lack information on specific epitopes.

Array technologies can help to fill this gap and identify epitopes that are targeted by antibodies, which in turn may be used to support the development of vaccines or monoclonal antibodies as therapeutics. High-density peptide arrays enable the rapid identification of antigen epitopes recognized by antibodies for many applications [9]. Pathogen-specific peptide arrays help to identify biomarkers for (early) detection of diseases [10]. Glycan arrays allow for the characterization and surveillance of viruses, identification of biomarkers, profiling of immune responses to vaccines, and epitope mapping [11,12]. 

In this study, we evaluate three distinct assays to identify the development of SARS-CoV-2 specific antibodies: (i) peptide arrays, covering the whole SARS-CoV-2 proteome as overlapping linear peptides, (ii) glycan arrays with a selected glycan library, and (iii) spike glycoprotein ELISA. We assess the ability of these assays to identify distinct epitopes, which can serve as potential biomarkers for disease progression. In combination with the clinical data of patients, we gained insights into immunoglobulin A, G, and M (IgA, IgG, and IgM) responses during COVID-19 progression.

Here, we report longitudinal antibody response data from three SARS-CoV-2-positive patients, sampled three times. While patient #1 had a moderate course of disease and was hospitalized (no ventilation), patient #2 experienced mild symptoms. Patient #3, who also had mild symptoms, was sampled only twice during disease and once 180 days before infection, which served as the negative control. Finally, for comparison, we added one sample of a single time point from another COVID-19 patient #4.

## 2. Materials and Methods

### 2.1. Patient Material

Blood samples were collected at the University Medical Center Hamburg-Eppendorf and the serum was immediately separated at 2000 g for 10 min, aliquoted, frozen, and stored at −80 °C. Table 1 lists information on patients and blood collection days. 

### 2.2. Serum IgA, IgG, and IgM Elisa

Semi-quantitative SARS-CoV-2 IgA, IgG, and IgM enzyme-linked immunosorbent assay (ELISA) targeting the S1-Domain of the S-spike protein subunit were performed (Euroimmun AG, Lübeck, Germany) according to the manufacturer’s instructions. Optical density was determined at a wavelength of 450 nm (OD450) and correction set to 620 nm. Ratios were calculated by Ratio = (Extinction control or sample)/(Extinction calibrator). A calibrator and positive control were provided with each ELISA kit. According to the manufacturer, a ratio of ≥1.1 should be regarded as positive and the manufacturer reports a specificity of 92.5 % for IgA, 99.3 % for IgG, and 98.6 % for IgM.

### 2.3. Peptide and Glycan Microarrays

The whole proteome of SARS-CoV-2 (GenBank ID: MN908947.3) was mapped as 4883 spots of overlapping 15-mer peptides with a lateral shift of two AA on peptide microarrays, obtained from PEPperPRINT GmbH (Heidelberg, Germany). Glycan microarrays containing a selection of 135 glycans were produced at CIC biomaGUNE (San Sebastián, Spain) [13]. Patient sera were diluted 1:200 (peptide) or 1:100 (glycan) and incubated on the arrays overnight. Afterwards, IgG, IgM, and IgA serum antibody interactions were differentially detected with fluorescently labeled secondary antibodies. For details, see Supplementary Materials.

## 3. Results

We collected blood of COVID-19 patients at different time points (Table 1) and used ELISA, peptide, and glycan microarrays to evaluate the kinetics of antibody development in detail.

Patient #1, a 64-year-old male, developed general weakness, myalgia and headache, intermittent episodes of very high fever, and subsequently, a productive cough. Two days after the first symptoms, he was tested positive for SARS-CoV-2 by RT-PCR. At that time point, the fever had already subsided, but a low-grade temperature recurred in the second week. An increase of C-reactive protein (53 mg/dL) required oral treatment with beta-lactamase antibiotic. The patient was hospitalized for four days and showed moderate but typical ground glass opacities on a high-resolution thorax computed tomography scan; he fully recovered without ventilation support. The patient did not require intensive care treatment or ventilation and the symptoms were moderate, due to hospitalization. Patient #2, his wife, a 62-year-old female, tested SARS-CoV-2 positive six days after her husband’s first symptoms. She had high viral shedding of SARS-CoV-2 monitored by RT-PCR, although she reported only very mild clinical symptoms of COVID-19, such as sub-febrile temperatures, a mild cough, and a constant sense of well-being, as stated by Pfefferle and colleagues [14]. Patient #3 tested positive for SARS-CoV-2 with a mild course of disease, without hospitalization. Since this participant donated serum on a regular basis, a serum sample was collected 180 days before the emergence of SARS-CoV-2, serving as a negative control. In addition, one sample served as another SARS-COV-2 positive control (#4, single time point d12, mild symptoms, see Supplementary Materials).

**Figure 1:**
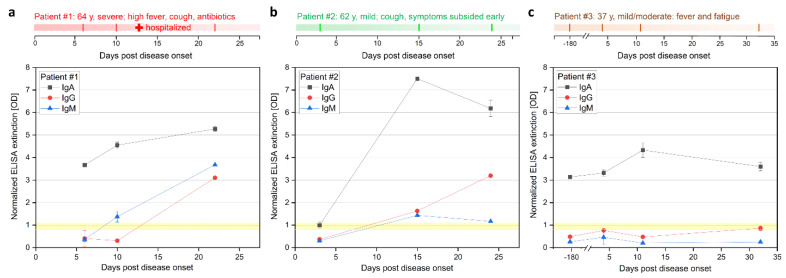
Longitudinal IgA, IgG, and IgM ELISA antibody response during COVID-19 disease progression in three patients. (**a**) Patients #1 (moderate), (**b**) #2 (mild), and (**c**) #3 (mild case). Patient #3 was also sampled 180 days prior infection, serving as a control. Sample from #3 collected at d32 was only analyzed with ELISA. ELISA (Euroimmune) was performed with S GP subunit 1 for detection of IgA and IgG at different days after onset of symptoms. Positive signal >1.1, negative signal <0.8, and intermediate 0.8–1.1 (highlighted yellow).

To evaluate the kinetics of B-cell epitopes during the mild and moderate courses of COVID-19, we first performed ELISA (EUROIMMUN, Lübeck, Germany) analysis (Figure 1, Supplementary A Table S1). This test relies on the S1 fragment of the spike glycoprotein (commercial test, likely AA1–685 of spike protein with glycosylation pattern). The IgG and IgM signals of patients #1 and #2 were below the threshold at early time points on day 6 (d6) and d3 respectively. The IgA signal of patient #1 was already highly positive on day 6 and increased until day 22. Patient #1 showed a positive signal for IgG only on day 22. The earliest positive results in patient #2 for IgG and IgM signals were measured on d15 and were also positive on d24, but only the IgG signal increased further over time. The IgA level of patient #2 showed a strong increase from the early time point d3 with an intermediate signal to the highest measured value on day 15. In patient #3, the assay failed to detect a clear positive IgG and IgM response, showing IgG signals in the intermediate level on days 4 and 32, while IgA appeared positive in all samples d-180, d4, d11 and d32 (for patient #4 see Supplementary Materials). 

Next, we applied full-proteome peptide microarrays (see Supplementary Materials B for complete peptide microarray data). Before we evaluated the SARS-CoV-2 specific signals, an antibody threshold signal had to be established. Here, we used the serum sample from donor patient #3 (healthy negative control) 180 days before SARS-CoV-2 infection as a negative control and defined the 99.9 th percentile fluorescence intensity (i.e., 5 out of 4883 signals considered false positive) as a threshold for positive IgA- and IgG-reactive peptides (IgA: 347.8 arbitrary fluorescence units (AFU) or 6.54 transformed AFU (tAFU); IgG: 1081.4 AFU or 7.68 tAFU). Our threshold selection successfully limited the amount of presented data for intelligibility, without losing precision, as we could confirm previously published epitopes (see Discussion).

**Figure 2:**
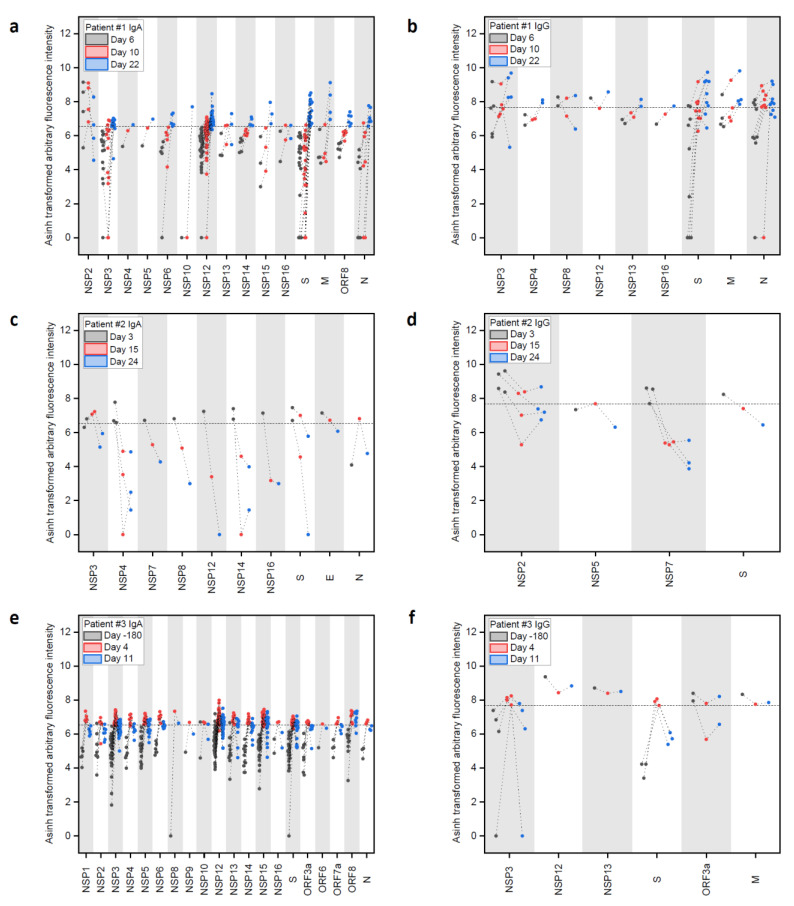
Longitudinal IgA and IgG antibody response against peptide epitopes above threshold from SARS-CoV-2 proteome during COVID-19 disease progression in three patients. (**a**–**f**) Evolution of positive IgA and IgG responses against SARS-CoV-2 peptides at different time points after onset of disease in patients #1, #2, and #3. Data were generated with peptide microarrays containing the whole SARS-CoV-2 proteome as 4883 overlapping peptides. Fluorescence intensities were transformed with the inverse hyperbolic sine (asinh function). Threshold selection shown as dashed horizontal lines: 99.9th percentile of IgA/IgG signals in healthy control sample #3 d-180, IgA: 6.54 transformed arbitrary fluorescence units, and IgG: 7.68 transformed arbitrary fluorescence units. For full array data, see Supplementary Materials B.

As a general trend, we observed SARS-CoV-2 protein-specific IgA and IgG responses with few defined signals, while IgM showed more signals, but without a clear trend. Thus, we focused on IgA and IgG responses. The evolution of IgA and IgG antibodies, targeting peptides of the SARS-CoV-2 proteome in patients #1 (Figure 2a,b) and #2 (Figure 2c,d) showed different dynamics: The moderate case (patient #1) showed a strong increase in IgA- and IgG-reactive peptides (above the control sample threshold) over time and eventually targeting many more epitopes (Supplementary Materials A Table S2). In comparison, the mild cases (patients #2 and #3) had a higher number of IgG- and an even higher number of IgA-reactive SARS-CoV-2 peptides already at d3 and d4, respectively, post onset of symptoms, which decreased over time (Supplementary Materials A Table S2). At these early time points, we already detected IgA and IgG-specific epitopes in the spike protein in patients #2 and #3, while patient #1 developed a high number of antibodies targeting spike epitopes only later at d22.

Patient #1 showed IgA responses at d6 (Figure 2a and Supplementary Materials A Table S2), solely targeting NSP2. The response was still limited four days later (d10), but then developed into a broad response at d22, targeting the nonstructural (NSP), the spike (S), membrane (M), ORF8, and nucleocapsid (N) proteins. The number of identified IgA-specific epitopes found in the different proteins increased over time in patient #1, with a particularly strong response to NSP3 and NSP12, while three signals for NSP2 epitopes decreased considerably. Regarding IgG responses (Figure 2b), patient #1 developed antibodies targeting the S and M protein already at d6 and the number of detected epitopes increased until d22.

In comparison, patient #2 (mild case, Figure 2c) showed a stronger and more specific IgA response already at d3 against the S, E, N, and NS proteins, while the IgG response (Figure 2d) revealed binding to NSPs and S. Patient #3 showed a strong and early response in IgA against many NSPs and the S protein (Figure 2e) comparing d-180 and d4, while the IgG (Figure 2f) only showed an increase in binding to NSP3 and S.

**Figure 3:**
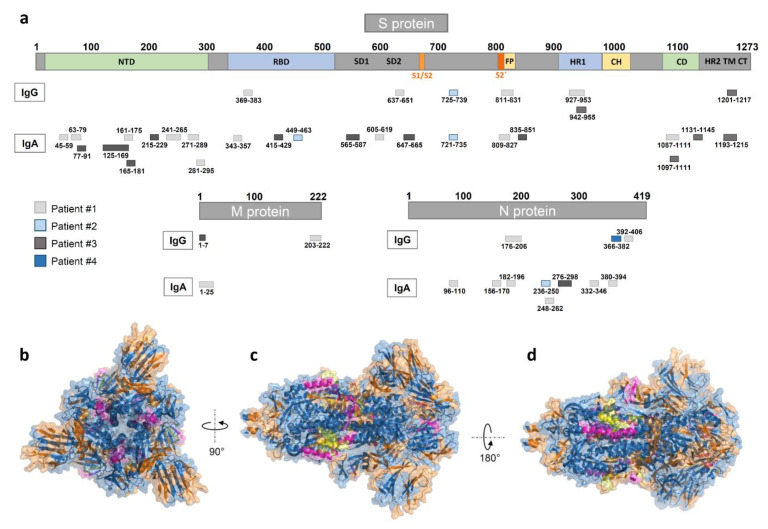
Mapping of peptide microarray reactive antibodies on the S, M, and N-proteins. (**a**) IgG and IgA epitopes derived from all four patients on the S (domains from [15]), M, and N proteins (see Supplementary Materials A Table S3). (**b**–**d**) A 3D view of the S GP structure (in blue) with the herein identified IgA (highlighted in orange), IgG (highlighted in magenta), and overlapping (highlighted in yellow) epitopes derived from all nine SARS-CoV-2 patient samples (generated with PyMOL from Protein Data Bank file 6vxx—cryo-EM structure of S GP [16]). (**b**) Top view, (**c**) side view, and (**d**) alternate side view of S GP trimer.

We visualized the identified epitopes derived from all patients (nine patient samples vs. control sample #3 d-180) on the S, M, and N proteins (Figure 3 and Supplementary Materials A Table S3). The data showed generally more IgA than IgG or IgM epitopes, with most epitopes located in the S and N protein.

**Figure 4:**
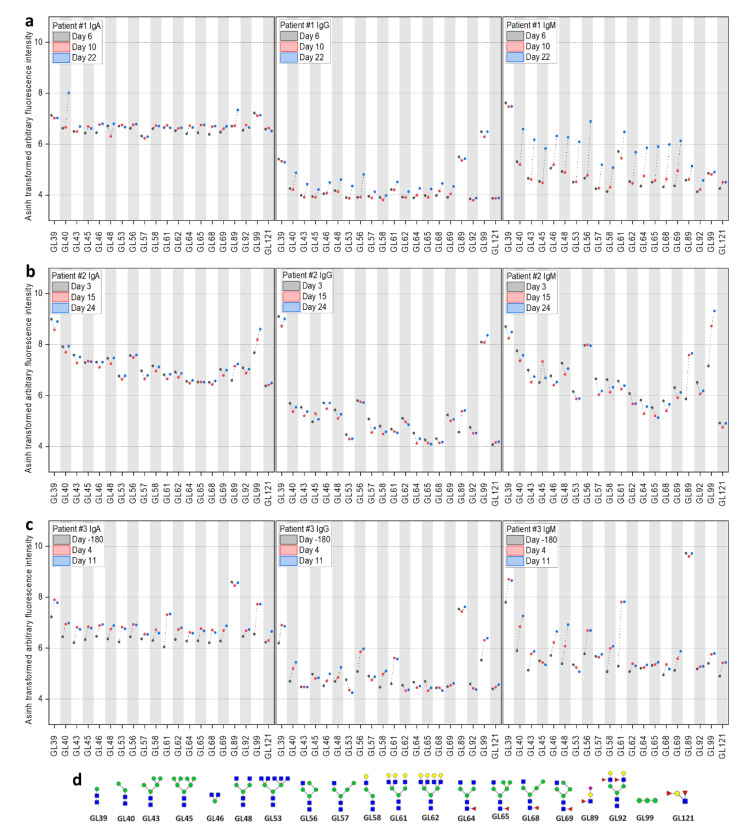
Longitudinal IgA, IgG, and IgM antibody response analyzed with glycan microarrays during COVID-19 disease progression in three patients. Evolution of reactive IgA, IgG, and IgM antibodies in patients #1–#3 (**a**–**c**) against a selection of glycans (**d**) on the microarrays at different time points (see Supplementary Materials C). For improved visualization, fluorescence intensities were transformed with the inverse hyperbolic sine (asinh function).

Finally, serum samples were analyzed with glycan microarrays, covering a diverse library of glycans (Figure 4, detailed information in Supplementary Materials C and Supplementary Materials A Figure S1). The glycans on the arrays cover several epitopes of the glycan shield of the SARS-CoV-2 surface [17]. Strong binding could be observed for the *N*-glycan core fragment (Man_2_GlcNAc_2_). Furthermore, α1-2-Man_3_ showed increased binding in convalescent time points, which hints to binding of high-mannose (M7–M9) structures, which are reported to be part of the glycan shield of SARS-CoV-2 (especially spike N234) [17]. Similar to the observed trend with the peptide microarrays, patient #1 showed a strong increase in antibody binding at d22 towards the *N*-glycan core structures (strongest increase observed in IgM), while patient #2 had generally stronger and more constant signals, except for the binding to α1-2-Man_3_. These results confirmed the general trends observed in both peptide and glycan microarray approaches, showing a strong increase of antibody responses in patient #1 at d22.

## 4. Discussion

To better understand the development of antibodies in SARS-CoV-2 infection and their consequence on the course of disease, we evaluated three assays that monitor the kinetics of B-cell epitope development in relation to clinical features. Three patients were sampled longitudinally. Two patients experienced a mild disease course (#2 and #3), another a moderate course (#1). We compared the longitudinal antibody response data using spike glycoprotein ELISA, peptide arrays of the whole SARS-CoV-2 proteome, and glycan arrays.

Serology testing for COVID-19 using ELISA is attractive because of the relatively short time to diagnosis and the ability to test for an active immune response against the virus. Comparing the three different approaches, the ELISA gives rather robust signals in the convalescent phase, while failing for early antibody detection. It is possible that the ELISA detects antibodies that either bind conformational/discontinuous epitopes or to glycopeptides. The assay gave similar positive results for both patients #1 and #2 in the convalescent phase (days 22 and 24). Comparing the data of patient #2 with mild symptoms we found that the peptide array shows a decline of binding to linear peptides over time, while the ELISA points into the opposite direction. In contrast, patient #1, with moderate symptoms, shows much stronger signals to the linear peptides on the array over time, which corresponds to the ELISA data. However, ELISA was inconclusive for patient #3, resulting in generally positive IgA and generally negative (or intermediate) IgG results for all time points, including d-180 prior infection and d32 (only analyzed by ELISA).

Recently, early antibody responses have been reported by ELISA [18], where seroconversion was found on day 7 after onset of symptoms in 50% of analyzed individuals. Another study underlined the early responses of IgA, IgM, and IgG following SARS-CoV-2 infection [19]. The authors reported a median duration of IgM and IgA antibody detection of five days and the detection of IgG 14 days after disease onset. Furthermore, Okba et al. [8] analyzed IgA and IgG responses in two mild and one moderate case using an in-house S1-ELISA. They observed an increase in the IgA response over time in a moderate case. An early or increased IgA response on arrays, as seen in our patients #2 and #3, was not observed with ELISA, possibly due to differences in the patients (sample collection dates) or assay performance. Key differences in these assays are the limitation of only using S1-proteins for the ELISA (vs. whole proteome on the microarray) and a higher sensitivity of peptide arrays towards linear epitopes. With the peptide arrays, cross-reactions to previous infections (e.g., with other coronaviruses) may become visible.

In contrast to ELISA, arrays are more time- and cost-intensive but provide more information on the development of antibodies. We identified several spike protein epitopes that are bound by IgA antibodies. We identified spike-specific IgA epitopes in the receptor binding domain, AA343-357, AA415-429, and AA449-463. The latter epitope is located in the receptor binding domain-angiotensin-converting enzyme II (RBD-ACE2)-complex and, therefore, may be the target of neutralizing antibodies [20]. In addition, we also confirm a part (AA369-383) of the SARS-CoV-2 and SARS-CoV cross-reactive IgG epitope (AA369-392) identified by Yuan et al., which is located in the receptor binding domain of spike [21]. Next, we observed IgA (AA809-827) and IgG (AA811-831) antibody binding, corresponding to the S2 cleavage site and fusion peptide. These have been described as distinctive epitopes in COVID-19 patients with neutralizing potential [20,22]. Furthermore, we could identify reactive peptides, especially in the N protein, as well as NSP3 and NSP12. Data from a partial proteome array approach was reported [23], which confirms strong binding to the N protein, although they did not cover NSP3 and NSP12. In contrast to NSP-binding antibodies, which could be cross-reactive from other viral infections, antibodies binding structural proteins like the S and N proteins, could be more distinctive for a SARS-CoV-2 infection [22]. It will be of interest to determine the longevity of these antibody responses and its impact on neutralization [24]. 

With the peptide arrays, we detected an early IgA response in the mild cases (patients #2 and #3). Respiratory viruses can induce efficient IgA responses in secretions as well as in sera. It was proposed that an early IgA response is predominant in COVID-19 and is more effective in SARS-CoV-2 neutralization than IgG [25]. IgA antibodies might be valuable diagnostic markers for early SARS-CoV-2 identification especially in mild-symptom patients. Due to high sensitivity and specificity, arrays may be relevant as diagnostics for the detection of these early antibody responses. Patient #2 potentially benefited from her early IgA response, which led to a mild course of the disease.

Employing glycan arrays, we identified several glycans that correspond to small fragments of the *N*-glycan core (e.g., Man_2_GlcNAc_2_). In addition, we observed an increase in binding to α1-2-Man_3_ (GL99 on the array) in patients #2 and #3. This fragment is part of the antennae of high-mannose (M7–M9) *N*-glycans, present on the spike protein (e.g., N122, N234, N343, and possibly others) [17,26]. A promising, but technically overly challenging approach, would be to screen glycopeptides with native glycan structures. Casalino et al. highlighted the modulating role of the spike protein N-glycan sites N165 and N234 for the conformation of the RBD [27]. Furthermore, a neutralizing antibody has been identified that binds a larger glycopeptide epitope of the SARS-CoV-2 spike protein [28]. Interestingly, we observe many spike-related peptide epitopes on the array, which would carry an *N*-glycosylation on the native virus (e.g., patient #1 in spike: AA63-79, AA271-289, AA343-357, AA605-619, and AA1087-1111). The glycan arrays generally show a similar trend as the peptide array results: the antibody response increases in patient #1 over time, whereas it stays constant or decreases over the course of the infection in patients #2 and #3, except for α1-2-Man_3_. Since many microorganisms express α1-2-Man_3_ on their surface, the SARS-CoV-2 infection might have caused a boost of a pre-existing immune response towards this epitope. Yet, data has to be evaluated in a broader context, since signals to glycans may be part of an unrelated cross-reaction or response to a larger glycopeptide epitope and multivalency can strongly influence the results.

We screened longitudinal serum samples of COVID-19 patients with different methods to get insights into their antibody responses and compared our data with findings of recent literature. A clear limitation of our study is the number of subjects, but still we were able to observe trends for the development of antibodies early after SARS-CoV-2 infection. Since all samples were collected from the same cluster of infection, which were the first detected SARS-CoV-2 infections in Hamburg, Germany, a clear chain of infection could be assured and samples could be collected repeatedly. This, and the limited access to arrays (especially glycan arrays), restricted the cohort size.

Our study emphasizes the importance of microarrays for early diagnostics and understanding of antibody development following SARS-CoV-2 infection. Arrays are able to reveal heterogeneous antibody responses in patients with different severity of symptoms. With a high assay sensitivity, antibody development in patients can be tracked during the course of disease and also early after infection. A general limitation of arrays is the use of exclusively linear peptides, which cannot identify antibodies that bind conformational or discontinuous epitopes. We exclusively considered the initially published Wuhan strain without mutations, but can quickly incorporate these mutations into the assay, since the array production method is rapid and flexible [29]. 

With the limitations listed above, our study contributes to the understanding of differences in the course of disease. There is still limited understanding of the immune correlates of protection. Collectively, we present an analysis of longitudinal antibody response in serum samples, comparing the degree of disease severity with three different approaches.

## Figures and Tables

**Table 1:** Patient and serum sample information; samples analyzed with peptide and glycan microarrays.

Sample	Patient ID	Gender	Age [y]	Symptoms	Hospitalized	Day of Serum Collection after Onset of Symptoms
1						d6
2	#1	Male	64	Moderate	Yes	d10
3						d22
4						d3
5	#2	Female	62	Mild	No	d15
6						d24
7						d-180
8	#3	Male	37	Mild	No	d4
9						d11
10	#4	Female	23	Mild	No	d12

## Data Availability

The data that support the findings are available online as supporting information or upon reasonable request from the corresponding authors.

## Supplementary Materials

The following are available online at https://www.mdpi.com/article/10.3390/pathogens10040438/s1. Supplementary A: Additional Information; Supplementary B: Peptide Microarray Data; Supplementary C: Glycan Microarray Data.
