# Supplementary material for: Longitudinal Development of Antibody Responses in COVID-19 Patients of Different Severity with ELISA, Peptide, and Glycan Arrays: An Immunological Case Series
Source: Pathogens. 2021 Apr 6;10(4):438. doi: 10.3390/pathogens10040438 (PMC8067489; doi:10.3390/pathogens10040438)
Supplement: Supplementary file 1 [file pathogens-10-00438-s001.zip › pathogens-1149654-supp/Supporting_Information_A_17_review_clean.docx]

**Supporting Information**

**Longitudinal development of antibody responses in COVID-19 patients of different severity with ELISA, peptide, and glycan arrays: an immunological case series**

Christine Dahlke, Jasmin Heidepriem, Robin Kobbe, René Santer, Till Koch, Anahita Fathi, Bruna M. S. Seco, My L. Ly, Stefan Schmiedel, Dorothee Schwinge, Sonia Serna, Katrin Sellrie, Niels-Christian Reichardt, ID-UKE COVID-19 study group, Peter H. Seeberger, Marylyn M. Addo, Felix F. Loeffler

*Enzyme linked immunosorbent assay – ELISA*

ELISA of the S1 subunit of spike glycoprotein (EUROIMMUN Medizinische Labordiagnostika AG, https://www.euroimmun.com) was used to analyze antibody titers of IgA, IgG, and IgM in patients. The assay was performed according to the manufacturer’s protocol. Optical density (OD) was detected at 450 nm and we calculated a ratio of the reading of each sample to the reading of the included calibrator for each sample (normalized OD). A positive signal is assumed for an OD above 1.1 and a negative signal below 0.8, whereas a signal between 0.8 – 1.1 is considered as intermediate.

**Table S1.** Normalized extinction [OD] of ELISA. Classification: negative < 0.8,

intermediate 0.8 – 1.1, and positive > 1.1.

| **Sample** | **IgA** | **IgG** | **IgM** |
| --- | --- | --- | --- |
| Positive control | 3.7 | 2.4 | 3.0 |
| Negative control | 0.5 | 0.5 | 0.2 |
| Patient #1 d6 | 3.7 | 0.4 | 0.4 |
| Patient #1 d10 | 4.6 | 0.3 | 1.4 |
| Patient #1 d22 | 5.3 | 3.1 | 3.7 |
| Patient #2 d3 | 1.0 | 0.4 | 0.3 |
| Patient #2 d15 | 7.5 | 1.6 | 1.4 |
| Patient #2 d24 | 6.2 | 3.2 | 1.2 |
| Patient #3 d-180 | 3.1 | 0.5 | 0.3 |
| Patient #3 d4 | 3.3 | 0.8 | 0.5 |
| Patient #3 d11 | 4.3 | 0.5 | 0.2 |
| Patient #3 d32 | 3.6 | 0.9 | 0.2 |
| Patient #4 d12 | 3.2 | 0.8 | 0.7 |

*Peptide microarrays*

The whole proteome of SARS-CoV-2 (GenBank ID: MN908947.3) was mapped as overlapping sequences on peptide microarrays. The sequences of ORF1ab polyprotein, spike glycoprotein (S), ORF3a protein, envelope protein (E), membrane glycoprotein (M), ORF6 protein, ORF7a protein, ORF8 protein, nucleocapsid phosphoprotein (N), and ORF10 protein were elongated and linked by neutral GSGSGSG linkers to avoid truncated peptides. The elongated protein sequences were translated into 4 883 different 15 amino acid peptides printed in duplicate (9 766 peptide spots) with a peptide-peptide overlap of 13 amino acids for high resolution epitope data. Peptide microarrays were obtained from PEPperPRINT GmbH (Heidelberg, Germany). Regarding the reproducibility of the peptide microarray assay, according to the vendor PEPperPRINT GmbH, the coefficient of determination between the batch control array of our microarray batch (all our arrays were from the same batch) and the general standard control microarray was R² = 0.888. Since all our peptide arrays were from the same batch, the intra-batch R² is expected to be > 0.9. To estimate the limit of detection (LoD), blank arrays were incubated with all secondary antibodies, anti-human IgG Dylight680 (Bethyl, A80-304D6) 0.5 mg/mL 1:1000, anti-human IgA Dylight800 (Rockland, 609-145-006) 1.0 mg/mL 1:1600, anti-human IgM Dylight 549 (Rockland, 609-142-007) 1.0 mg/mL 1:2000, control anti-HA (BioXcell, RT0168) 1.0 mg/ml labeled 680 1:2000. The secondary antibodies show negligible (unspecific) binding to the slide surface (< 50 AFU). The LoD was calculated according to Armbruster & Pry, 2008.^[1]^ Using the blank control (= staining only with secondary antibodies), in the traditional approach, the LoD is calculated as the mean of blank control +2x standard deviation. This results in estimations for LoD IgG (700nm) = 134 AFU + 2x 36 AFU = 206 AFU, LoD IgA (800nm) = 118 AFU + 2x 48 AFU = 214 AFU, LoD IgM (532nm) = 177 AFU + 2x 59 AFU = 295 AFU. However, the LoD of the assay is impacted by its semi-quantitative nature of screening many interactions in parallel, which include false positive hits, as well as cross-reactive epitopes. Therefore, we chose to use the negative control serum as the background, to reduce the impact of false positive and cross-reactive epitopes.

*Glycan microarrays*

Glycan microarrays were prepared as previously described.^[2]^ Briefly, solutions of aminopentyl functionalized carbohydrates (50 μM, 1.25 nL, 5 drops, drop volume: 250 pL) in printing buffer (300 mM sodium phosphate + 0.005 % Tween® 20) were arrayed on N-hydroxysuccinimide (NHS) activated glass slides (Nexterion® slide H, Schott AG) employing a piezoelectric non-contact printer (SciFLEXARRAYER S11, Scienion). Each carbohydrate was printed in 4 replicates, generating 7 identical subarrays per slide. After printing, slides were incubated at humidity over 75 % overnight at RT. After immobilization, the remaining NHS groups were quenched (50 mM ethanolamine in sodium borate buffer 50 mM pH = 9) at RT for one hour. To estimate the limit of detection of the glycan arrays, we incubated the arrays only with the secondary antibodies, to have a blank control value for the detection without sample: Anti-human IgG Alexa Fluor 647 (SouthernBiotech, 2048-31) 0.5 mg/ml 1:400, anti-human IgA Rhodamine (Rockland, 609-1006) 2.0 mg/ml 1:400, anti-human IgM Alexa Fluor 594 (Invitrogen, A21216) 2.0 mg/ml 1:400. Again, LoD was calculated according to Armbruster & Pry, 2008.^[2]^ Using the blank control, the LoD is calculated as the mean of blank control +2x standard deviation of the blank control (= staining only with secondary antibodies). This results in LoD IgG (635 nm) = 30 AFU + 2x 51 AFU = 132 AFU, LoD IgA (532 nm) = 258 AFU + 2x 85 AFU = 428 AFU, LoD IgM (594 nm) = 53 AFU + 2x 64 AFU = 181 AFU. Due to the higher autofluorescence (glass, coating, etc.) in this wavelength, the IgA signal has a higher LoD signal.

*Processing and analysis of peptide microarrays*

Before incubation of the serum samples, the arrays were pre-swollen for 15 min with 1.6 mL PBST (0.05 % (v/v) Tween 20 in PBS) at room temperature and orbital shaking at 100 rpm. To avoid nonspecific binding of the serum proteins, the arrays were incubated with blocking buffer (MB-070, Rockland Immunochemicals Inc., Limerick, USA) for 30 min at 100 rpm and room temperature. After short washing with PBST, 1.6 mL of sera, diluted 1:200 in staining buffer (10 % (v/v) blocking buffer in PBST), were incubated overnight at 100 rpm and 4 °C. To remove unbound serum components, the arrays were washed quickly three times with PBST. The human serum antibody classes IgG, IgM and IgA were detected with fluorescently labeled secondary antibodies: 0.5 mg/ml anti-human IgG-Fc fragment cross-adsorbed DyLight 680 conjugated (A80-304D6, Bethyl Laboratories, Montgomery, USA), 1.0 mg/ml anti-human IgM (mu chain) DyLight 549 conjugated (609-142-007, Rockland Immunochemicals Inc., Limerick, USA), 1.0 mg/ml anti-human IgA (alpha chain) antibody DyLight 800 conjugated (609-145-006, Rockland Immunochemicals Inc., Limerick, USA). The HA control peptides on the array were detected with 1.0 mg/ml anti-HA-peptide antibody (RT028, Bio X Cell, New Hampshire, USA) labeled with Lightning-Link Rapid Dylight 680 (327-0010, Innova Biosciences Ltd., Cambridge, United Kingdom). The secondary antibodies were diluted in staining buffer (anti-human IgG 1:1 000, anti-human IgA 1:1 600, anti-human IgM 1:2 000 and anti-HA-peptide 1:2 000) and applied to the microarrays for 30 min at 100 rpm at room temperature in the dark. To remove unbound secondary antibodies, the arrays were washed quickly three times with PBST. Finally, the arrays were dipped in 1 mM Tris HCl pH 7.4 and dried in a jet of air. The arrays were scanned and fluorescence signals were detected at 700 nm and 800 nm using an Odyssey Scanner (LI-COR Biotechnology Inc., Lincoln, Nebraska, USA) and at 532 nm with a Genepix 4000B (Molecular Devices, San José, USA). Analysis of the scans was performed using PepSlide Analyzer software (SICASYS Software GmbH, Heidelberg, Germany). For quantification, the fixed-spot detection method was used, the intensity values were derived as aggregate foreground median values (the intensity median of two spot duplicates was calculated), while the local spot background was subtracted.

*Processing and analysis of glycan microarrays*

To avoid nonspecific binding of the serum proteins, the arrays were incubated with 1 % (w/v) BSA in PBS for 60 min at 120 rpm and room temperature. After three times washing with PBS, 200 µL of sera, diluted 1:100 in 1 % (w/v) BSA in PBS, were incubated overnight at 120 rpm and 4 °C. To remove unbound serum components, the arrays were washed quickly three times with PBS. The human serum antibody classes IgG, IgM and IgA were detected with fluorescently labeled secondary antibodies: 0.5 mg/ml anti-human IgG Fc Alexa Fluor 647 (2048-31, SouthernBiotech, Birmingham, USA), 2.0 mg/ml anti-human IgA (alpha chain) Rhodamine (609-1006, Rockland Immunochemicals Inc., Limerick, USA), 2.0 mg/ml anti-human IgM (Heavy chain) Alexa Fluor 594 (A21216, Invitrogen, Carlsbad, USA). The secondary antibodies were diluted 1:400 in 1 % (w/v) BSA in PBS and applied to the microarrays for 60 min at 120 rpm at room temperature in the dark. To remove unbound secondary antibodies, the arrays were washed quickly three times with PBST. Finally, the arrays were dipped in water and dried in a jet of air. The arrays were scanned and fluorescence signals were detected at 532 nm, 594 nm, and 635 nm using a GenePix 4300A (Molecular Devices, San José, USA). Analysis of the scans was performed using PepSlide Analyzer software (SICASYS Software GmbH, Heidelberg, Germany).

**Table S2.** Number of reactive SARS-CoV-2 peptides at different time points targeted by IgA, IgG, and IgM antibodies above the threshold for fluorescence intensity (99.9^th^ percentile IgA: 347.8 A.F.U.; 99.9^th^ percentile IgG: 1081.4 A.F.U.; 99.5^th^ percentile IgM: 4239.1 A.F.U.) of patients #1 – #4. Data derived from peptide microarray data, showing hits for ORF1ab polyprotein, spike glycoprotein, and all proteins.

**Table S3.** Positive SARS-CoV-2 peptides in IgG, IgA, and IgM response for the spike glycoprotein, membrane glycoprotein, and nucleocapsid phosphoprotein.

| **Spike glycoprotein (S)** | | | | | |
| --- | --- | --- | --- | --- | --- |
| **IgG** | | **IgA** | | **IgM** | |
| Position | Peptide | Position | Peptide | Position | Peptide |
|  |  | 45-59 | SSVLHSTQDLFLPFF | 45-59 | SSVLHSTQDLFLPFF |
|  |  | 63-91 | TWFHAIHVSGTNGTKRFDNPVLPFNDGVY |  |  |
|  |  | 125-181 | NVVIKVCEFQFCNDPFLGVYYHKNNKSWMESEFRVYSSANNCTFEYVSQPFLMDLEG |  |  |
|  |  | 215-229 | DLPQGFSALEPLVDL |  |  |
|  |  | 241-265 | LLALHRSYLTPGDSSSGWTAGAAAY | 241-265 | LLALHRSYLTPGDSSSGWTAGAAAY |
|  |  | 271-295 | QPRTFLLKYNENGTITDAVDCALDP |  |  |
| 369-383 | YNSASFSTFKCYGVS | 343-357 | NATRFASVYAWNRKR |  |  |
|  |  | 415-429 | TGKIADYNYKLPDDF |  |  |
|  |  | 449-463 | YNYLYRLFRKSNLKP |  |  |
|  |  | 565-587 | FGRDIADTTDAVRDPQTLEILDI |  |  |
|  |  | 605-619 | SNQVAVLYQDVNCTE |  |  |
| 637-651 | STGSNVFQTRAGCLI | 647-665 | AGCLIGAEHVNNSYECDIP |  |  |
| 725-739 | EILPVSMTKTSVDCT | 721-735 | SVTTEILPVSMTKTS |  |  |
| 811-831 | KPSKRSFIEDLLFNKVTLADA | 809-827 | PSKPSKRSFIEDLLFNKVT |  |  |
| 927-955 | FNSAIGKIQDSLSSTASALGKLQDVVNQN | 835-851 | KQYGDCLGDIAARDLIC |  |  |
|  |  | 1087-1111 | AHFPREGVFVSNGTHWFVTQRNFYE |  |  |
|  |  | 1131-1145 | GIVNNTVYDPLQPEL | 1127-1141 | DVVIGIVNNTVYDPL |
| 1201-1217 | QELGKYEQYIKWPWYIW | 1193-1215 | LNESLIDLQELGKYEQYIKWPWY | 1143-1157 | PELDSFKEELDKYFK |
| **Membrane glycoprotein (M)** | | | | | |
| **IgG** | | **IgA** | |  | |
| Position | Peptide | Position | Peptide |  |  |
| 1-7 | MADSNGT | 1-25 | MADSNGTITVEELKKLLEQWNLVIG |  |  |
| 203-222 | NYKLNTDHSSSSDNIALLVQ |  |  |  |  |
| **Nucleocapsid phosphoprotein (N)** | | | | | |
| **IgG** | | **IgA** | | **IgM** | |
| Position | Peptide | Position | Peptide | Position | Peptide |
|  |  | 96-110 | GGDGKMKDLSPRWYF |  |  |
|  |  | 156-170 | AIVLQLPQGTTLPKG |  |  |
| 176-206 | SRGGSQASSRSSSRSRNSSRNSTPGSSRGTS | 182-196 | ASSRSSSRSRNSSRN | 182-196 | ASSRSSSRSRNSSRN |
|  |  | 236-262 | GKGQQQQGQTVTKKSAAEASKKPRQKR |  |  |
|  |  | 276-298 | RRGPEQTQGNFGDQELIRQGTDY |  |  |
| 366-382 | TEPKKDKKKKADETQAL | 332-346 | TYTGAIKLDDKDPNF |  |  |
| 392-406 | VTLLPAADLDDFSKQ | 380-394 | QALPQRQKKQQTVTL |  |  |


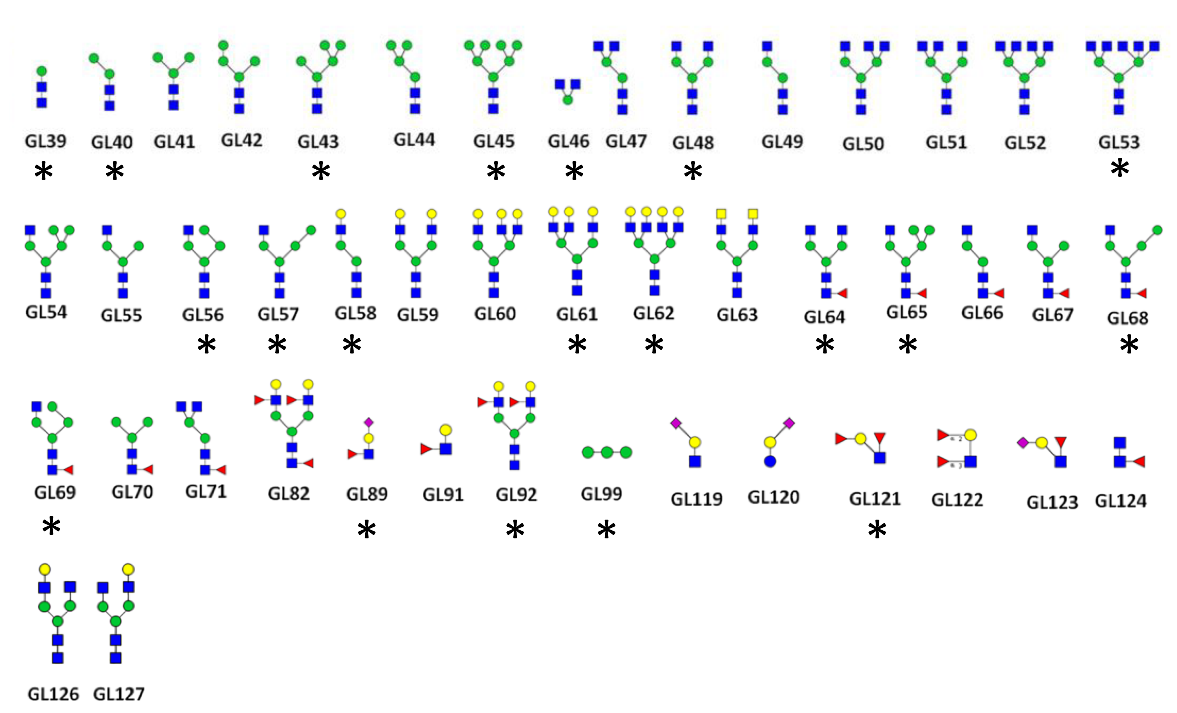


**Figure S1.** Glycans on the glycan microarrays (only human relevant structures of the in total 135 glycan structures are shown, see Supporting Information C for all glycans and data). Glycans with statistically significant signals are labeled with an asterisk (GL39, GL40, GL43, GL45, GL46, GL48, GL53, GL56–58, GL61, GL62, GL64, GL65, GL68, GL69, GL89, GL92, GL99, GL121).

**References**

1. Armbruster, D.A., and Pry, T. Limit of Blank, Limit of Detection and Limit of Quantitation. Clinical Biochemist Reviews, 2008. 29(Suppl 1): p. 49–52.

2. Echeverria, B., et al., Chemoenzymatic Synthesis of N-glycan Positional Isomers and Evidence for Branch Selective Binding by Monoclonal Antibodies and Human C-type Lectin Receptors. ACS Chemical Biology, 2018. 13(8): p. 2269-2279.
